# Dynamic Cancellation of Perceived Rotation from the Venetian Blind Effect

**DOI:** 10.3390/vision3020014

**Published:** 2019-04-03

**Authors:** Joshua J. Dobias, Wm Wren Stine

**Affiliations:** 1 Department of Psychology & Counseling, Marywood University, Scranton, PA 18509, USA; 2 Department of Psychology, University of New Hampshire, Durham, NH 03824, USA

**Keywords:** stereopsis, irradiation stereoscopy, Venetian blind effect, contrast disparity, luminance disparity, temporal dynamics, motion-in-depth, depth cancellation

## Abstract

Geometric differences between the images seen by each eye enable the perception of depth. Additionally, depth is produced in the absence of geometric disparities with binocular disparities in either the average luminance or contrast, which is known as the Venetian blind effect. The temporal dynamics of the Venetian blind effect are much slower (1.3 Hz) than those for geometric binocular disparities (4–5 Hz). Sine-wave modulations of luminance and contrast disparity, however, can be discriminated from square-wave modulations at 1 Hz, which suggests a non-linearity. To measure this non-linearity, a luminance or contrast disparity modulation was presented at a particular frequency and paired with a geometric disparity modulation that cancelled the perceived rotation induced by the luminance or contrast modulation. Phases between the luminance or contrast and the geometric modulation varied in 50 ms increments from −200 and 200 ms. When phases were aligned, observers perceived little or no rotation. When not aligned, a perceived rotation was induced by a contrast or luminance disparity that was then cancelled by the geometric disparity. This causes the perception of a slight jump. The Generalized Difference Model, which is linear in time, predicted a minimal probability in cases when luminance or contrast disparities occurred before the geometric disparities due to the slower dynamics of the Venetian blind effect. The Gated Generalized Difference Model, which is non-linear in time, predicted a minimal probability for offsets of 0 ms. Results followed the Gated model, which further suggests a non-linearity in time for the Venetian blind effect.

## 1. Introduction

Slight geometric differences between the images on each retina are often used to perceive depth within the environment. However, surfaces can also appear to rotate around a vertical axis when the image shown to one eye has lower average luminance or a Michelson contrast (Michelson contrast is defined as the difference between the maximum and minimum luminance values divided by the sum of the maximum and minimum luminance values, or (L_max_ − L_min_)/(L_max_ + L_min_) [1].) [2,3,4,5,6,7,8,9] than a geometrically-identical image shown to the other eye (Venetian blind effect). Blake and Cormack [10] failed to replicate the results of Fiorentini and Maffei [6].

### 1.1. Venetian Blind Effect

The Venetian blind effect has been explained as the creation of a geometric retinal disparity that results from the imperfect optics of the eye [2,3,4]. For a complete review, see Filley et al. [7], Dobias and Stine [9], and Larkin and Stine [3]. Cibis and Haber [4] state that a decrease in retinal illuminance causes a shift in the location at which the retinal illuminance of, for example, the bars of a 100% contrast square wave grating cross threshold. As a result, the above-threshold segments of the brighter bars are physically wider in the eye receiving higher average retinal illuminance than those in the eye viewing the lower average luminance. If so, a geometric disparity would exist between the two retinal images. A second possibility can be derived from von Helmholtz’s [11] notions concerning irradiation [1,2]. The imperfect optics of the eye [11] in combination with the compressive non-linearity in response to retinal illuminance causes the location of an edge to appear shifted toward the less intense side of that edge [11]. Galileo was familiar with the effect [12]. Westheimer [13] measured its magnitude to be about 0.4 minutes of arc when viewing a stimulus with a 113 cd/m^2^ bright region bordering a region of less than 1 cd/m^2^. von Helmholtz [11] did not discuss the Venetian blind effect, but his description of irradiation could be applied as a possible explanation for the effect. Again, an interocular difference in retinal illuminance would create a perceived geometric disparity due to the differential shift in the apparent location of the bar edges in the two eyes. Both theories would predict that, due to the increased apparent width of those bars in the higher-intensity image relative to that in the lower-intensity image, a square-wave grating viewed binocularly with an average luminance or contrast disparity would appear to have rotated bright bars [7].

Filley et al. [7] tested both irradiation theories. Results show that observers did report a perceived rotation when viewing gratings of less-than-unity contrast, which contradicts the model proposed by Cibis and Haber [4] since the model required the dark portions of the stimulus to be below threshold. Furthermore, when a blur between grating bars was introduced, which should increase the perceived shift in the location of bright bar edges [14,15,16,17,18,19], the threshold for perceived rotation and the amount of perceived rotation remained constant, which contradicts the irradiation model with a compressive nonlinearity [7]. Filley et al. [7] developed an intensity-difference model relating the interocular difference in retinal illuminance of the light bars of the grating to the probability of reporting rotation that did fit the observed data. The Venetian blind effect may help enhance the perceived rotation of distant surfaces by making small rotations more visible [9] (von Helmholtz [11] did not discuss the Venetian blind effect, but his description of irradiation could be applied as a possible explanation for the effect). Gillam and Wardle [20] argue that luminance disparities may disambiguate geometric disparity and/or slant viewed from eccentric directions. Therefore, perhaps the gating ensures that the enhanced perceived rotation is visible for static objects.

Hetley and Stine [8] introduced a geometric disparity by increasing the width of the bright bars seen by one eye and then introducing a disparity in luminance or contrast to cancel the perceived depth from the geometric disparity. With a sufficient disparity in contrast or luminance, results showed that perceived depth from luminance and contrast disparities did successfully cancel perceived depth from a disparity in width. Münster [2], Larkin and Stine [3] and Cibis and Haber [4] similarly found that a disparity in luminance was cancelled by a geometric disparity. This suggests that, at some level, information from each type of cue feeds into a common neural mechanism that then assigns a depth value. Of course, such a mechanism may take many forms. For example, geometric and luminance/contrast disparities may tap into distinct components that reciprocally inhibit one another.

The magnitude of perceived depth decreases as the temporal frequency of the geometric disparity modulations increase. Depth magnitude begins to decrease at frequencies between 1 and 2 Hz and falls below threshold at frequencies between 4 and 6 Hz [21,22,23,24,25,26,27]. Dobias and Stine [9] compared the critical frequency in the Venetian blind effect to that with a geometric disparity by altering contrast disparity cues at increasing frequencies. If the irradiation hypothesis correctly accounts for the Venetian blind effect, then an irradiation-produced width disparity at the retina should have the same critical frequency as a geometrically-produced width disparity. That is, irradiation states that a luminance or contrast disparity caused a geometric disparity and, therefore, the visual system should process perceived depth in similar ways for both disparity types. As a result, contrast disparity modulations should also lead to the perception of depth at frequencies of 5–6 Hz.

Dobias and Stine [9] dynamically altered the contrast of each grating in either a sine or square wave modulation. Each grating started at an equal contrast that was then increased in one eye as it was decreased in the other. The frequency of modulations was increased between 0.2 to 1.8 Hz. For sine wave modulations, results showed that the probability of reporting depth and motion-in-depth became less likely at frequencies above approximately 1 Hz and that depth was rarely reported when at approximately 1.8 Hz. Similarly, for square wave modulations, the probability of reporting depth again became less likely when above approximately 1 Hz, and was completely diminished before 1.8 Hz. As predicted, however, when the width of the grating bars was dynamically changed, depth alterations were visible at frequencies up to 5 Hz (in replication of References [21,22,23,24,25,26,27]).

Results from Dobias and Stine [9] show that the critical frequency for perceived depth from a geometric disparity is considerably greater than that from a contrast disparity. Again, irradiation models would predict that perceived depth from both a contrast and geometric disparity should have equal critical frequencies. Since perceived depth from a geometric disparity is visible at higher frequencies than depth from a contrast disparity, it is likely that both result from separate neural mechanisms at some level. In addition, since perceived depth from a contrast [8] and a luminance [2,3,4,8] disparity can be cancelled by that created using a geometric disparity, and perceived depth from contrast and luminance disparities can cancel one another [7], perceived depth from each type of cue is likely a result of activity in the same neural tissue. This assumption relies on the psychophysical linking hypothesis [28] that “…if two different stimuli (or their resulting sensations) are statistically indiscriminable, the corresponding neural signals must be rendered statistically indiscriminable somewhere within the sensory system, at or prior to the bridge locus” [29]. The bridge locus is the collection of “...central neurons that form the most immediate substrate of conscious perceptual events” [29].

Dobias & Stine developed two models to understand the dynamics of the Venetian blind effect, a Generalized Difference Model, and a Gated Generalized Difference Model (which are presented briefly in Appendix A, see Dobias and Stine [9], for a full description). Both models imagine a process where the generalized difference in contrast feeds into a common cyclopean locus. The two models differ, however, in the nature of that input. The Generalized Difference Model feeds contrast disparity into the cyclopean locus with a time constant that gives a critical frequency of about 1.3 Hz. With the gated model, however, the contrast disparity feeds into the cyclopean locus with a small time constant, which leads to a critical frequency in excess of 3 Hz. This input is gated by a process with a large time constant. Thereby, this constricts input when the frequency of the stimulus reaches about 1.3 Hz.

The un-gated model represents a linear system in time, which has strong implications for discriminating a sine-wave contrast disparity modulation from a square-wave modulation. A square wave with a given frequency and amplitude can be constructed as the sum of successive sine waves. The first sine wave in the sum will have a frequency equal to the original square wave. Each successive sine wave will have 3, 5, …, times the frequency and ⅓, ⅕, …, the amplitude of the first sine wave (e.g., Weisstein [30]). Assuming a critical frequency of about 1.3 Hz, a 0.25 Hz square wave should be discriminable from a 0.25 Hz sine wave by a linear system since, while they share the sine-wave component at 0.25 Hz, the square wave has an additional component at 0.75 (= 3 × 0.25) Hz, which is below the critical frequency and, therefore, should be visible. However, one should not be able to discriminate depth modulations from a sine-wave contrast disparity modulation from a square-wave modulation at frequencies above 0.43 (= 1.3/3) Hz since, at those frequencies, the second sine wave component in the square wave will have a frequency greater than the critical frequency. The non-linear gated model, on the other hand, enables the discrimination of perceived rotations induced by sine-wave modulations from those induced by square-wave modulations at frequencies of about 1 Hz while no perceived rotation occurs at frequencies above about 1.3 Hz.

### 1.2. Rationale for Current Research

The experiments conducted by Dobias and Stine [9] tested contrast disparities and geometric disparities separately while measuring the dynamics of perceived rotation. As described above, disparities in average luminance [4,8] and contrast [8] can cancel the perceived depth from a geometric disparity, which suggests that cues for each are fed into a common neural mechanism. Furthermore, the results of Filley et al. [7] and Dobias and Stine [9] suggest that the mechanisms feeding average luminance and the contrast information and geometric information into the common locus are not the same. Additionally, the mechanism feeding average luminance and contrast information into the common locus possesses a critical frequency on the order of 1.3 Hz and, yet, allows for the discrimination of a sine-wave temporal disparity modulation from a square-wave modulation at frequencies reaching 1.1 Hz, which suggests a radically non-linear system.

If one were to introduce a grating where the geometric disparity modulates in time but that modulates out of phase with the luminance or contrast modulation, it should be possible to set the phase lag so that the geometric disparity cancels the perceived rotation induced by the luminance or contrast disparity. A linear system, like the Generalized Difference Model, would predict that the lag required for cancellation would reflect the difference in time constants between the luminance or contrast channels and the geometric channel. The non-linear system described by the gated model, however, would predict that the required phase lag would be essentially zero since the two channels have similar time constants.

To measure these effects, a square-wave luminance or contrast modulation was presented at a particular frequency superimposed on a square-wave geometric disparity modulation that was chosen to cancel the perceived rotation induced by the luminance or contrast modulation. If the phases of the two modulations of perceived depth are properly aligned, observers should report little or no perceived rotation or depth during the modulations. However, if the phases are slightly misaligned, the observer should perceive a rotation induced by, for example, a contrast disparity that is then cancelled by the geometric disparity. A transient rotation, or jump, should be perceived during the interval when there is a perceived rotation from contrast disparity that has not yet been cancelled by the geometric disparity.

**Figure 1:**
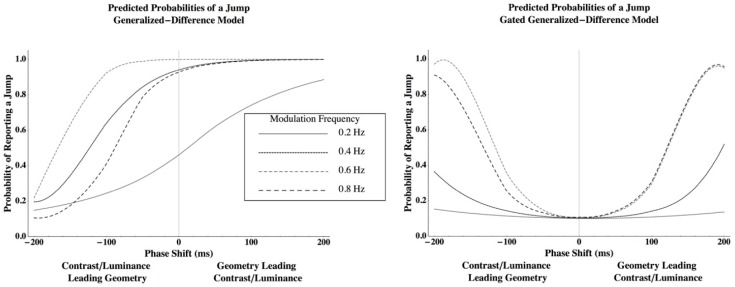
Predictions from the Generalized Difference Model (left) and the Gated Generalized Difference Model (right) when a geometric disparity modulation is used to cancel the perceived rotation induced by a contrast or average luminance disparity modulation. The probability of a jump corresponds to the probability of a brief rotation induced by one disparity before being cancelled by the second disparity. The details concerning the algorithm that was used to generate these predictions can be viewed in Appendix B.

Using this method, the Generalized Difference Model predicts that the probability of a perceived jump for relatively low frequencies will be minimized at a phase lag that is displaced from zero with the luminance or contrast disparity modulation, which leads the geometric modulation. The Gated Generalized Difference Model, however, predicts that the probability of a jump for relatively low frequencies will be minimized at a phase lag of nearly zero (Figure 1). The details concerning the algorithm that were used to generate these predictions are given in Appendix B.

## 2. Materials and Methods

### 2.1. Observers

Observers were PCN, JJD, and WWS. WWS and JJD were experienced observers. PCN was naive to specific experimental details and the expected results. All observers had normal or corrected to normal vision, had normal stereopsis, and provided informed consent before participation. All research was conducted in accordance with the Declaration of Helsinki and was approved by the University of New Hampshire Institutional Review Board (Approval Code: PS-110405A).

### 2.2. Apparatus

While sitting in a dark room, the observer’s head was stabilized by a bite bar individually molded for his teeth. To equalize the amount of light entering each eye, each observer viewed the stimuli through 3 mm apertures. Stimuli were presented on an Apple ColorSync display with a refresh rate of 75 Hz and viewed from a distance of 1.58 m. At this distance, one pixel subtended 38 s of the visual angle. A Mac Book Pro laptop running *Mathematica* 7.0.0 controlled the display. *Mathematica* was used to present animations and record responses.

**Figure 2:**
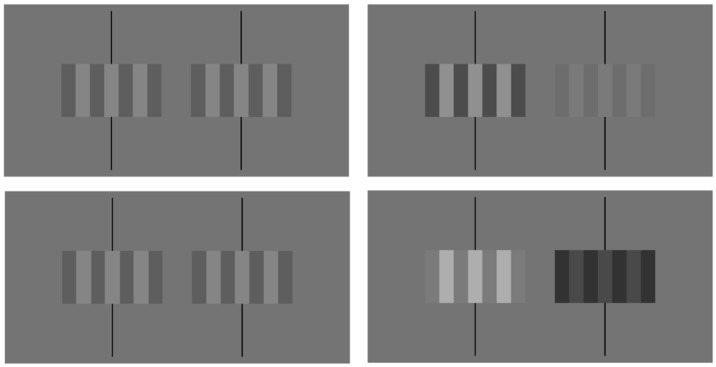
Stereograms with binocular fixation lines of rectangular-wave gratings that have zero disparity (top left), a geometric disparity created by wider bars on the left side grating (bottom left), a contrast (top right), or a luminance (bottom right) disparity corresponding to a dichoptic contrast modulation of approximately 0.6. Either crossed or uncrossed fusion is appropriate. The bars of the zero disparity stimulus (top left) should appear to be in the fronto-parallel plane. For the other three stimuli that contain disparities (geometric, contrast, and luminance), the lighter bars of the static stimuli will appear to rotate with their right edges closer to the viewer. If crossed fusion is used, the lighter bars of the fused image will appear to rotate with their left edges closer to the viewer (see Reference [3] Figure 1 for a 100%-contrast example).

Luminance measures were taken with a Minolta LS-110 photometer. The maximum and minimum luminance values generated by the monitor were approximately 86 and 2 cd/m^2^, respectively. As a result, the largest average luminance value that could be presented was 44 cd/m^2^ and the maximum contrast value that could be presented was approximately 0.95. For experimental trials, the baseline average luminance value for each grating was set at 29 cd/m^2^ with a baseline contrast of 0.325. The contrast or the luminance of the gratings (Figure 2) were either increased or decreased depending on experimental conditions. When the average luminance or contrast of the gratings were dynamically changed, the value of one quality (contrast or luminance) stayed constant while the other changed. For example, during a contrast condition, the average luminance of the grating viewed by each eye remained at 29 cd/m^2^ while the contrast of the grating viewed by one eye was increased to a value of 0.546 and decreased to a value of 0.104 when viewed by the other eye. Similarly, for luminance conditions, the contrast of the gratings viewed by each eye remained at 0.325 while the average luminance of the grating viewed by one eye was increased to 48.72 cd/m^2^ and the average of the grating viewed by the other eye was reduced to 9.28 cd/m^2^. Specific contrast, luminance, and geometric disparities were selected based on cancellation values determined by Hetley and Stine [8].

Each frame of the animation consisted of two square wave gratings placed side-by-side, which is similar to those in Figure 2. A baffle was placed vertically between the two gratings to ensure that the right side grating was visible only to the right eye and the left side grating to the left eye. Each grating consisted of four dark bars and three light bars with each of which was 1.577° in height and 0.410° in width. Each complete standard grating had a spatial frequency of 1.25 cycles per degree, and was 1.577° in height and 2.870° in width. The gratings had an overall average luminance of 29 cd/m^2^, which was equal to the luminance of the background, and an overall contrast of 0.325. Above and below each grating, 0.03° wide binocular fixation lines were placed to aid in the fusion of the two images. The center of each line contained a 0.137° by 0.515° vertical rectangle with the same luminance as each dark bar of the left grating.

To create an experimental stimuli, gratings were first altered to allow light bars presented to one eye to widen while those presented to the other eye remained the same. The left and right sides of each middle dark bar and one column of pixels of an end dark bar were replaced with a column of pixels that could be altered individually to be either lighter or darker. When the lighter bars viewed by one eye were altered to be wider, each individual light bar was 1.577° in height and 0.431° in width (Figure 2). The overall monocular image remained 1.577° in height and 2.870° in width. Second, a contrast disparity or average luminance disparity could be added to the stimuli. If the left eye viewed wider bars, it would also receive a grating with lower contrast or average luminance.

### 2.3. Procedure

In preparation for each experiment, observers sat in a chair facing the monitor while biting on a properly positioned bite bar to ensure a direct view of the monitor. An example stimulus was shown on the screen, and was viewed through an aperture that blocked all parts of the screen except for the stimulus.

Once the experiment began, the observer viewed a blank gray screen with a luminance of 29 cd/m^2^ for five minutes to adjust to the darkened room. After five minutes, a stimulus containing only binocular lines was presented for 10 s and then was replaced with an animated experimental stimulus that was presented for 10 s. After 10 s, the experimental stimulus was replaced with a blank gray screen, the observer was prompted to make a response, a response was made, and then the process began again (the five minute adaptation only occurred before the first trial).

Animations were generated and saved as *QuickTime 7* movie files. *Mathematica 7.0.0* then randomly chose one of the pre-made movie files, played the movie using *QuickTime 7*, prompted for a response, saved the response, and began the next trial. Contrast and luminance disparities were altered in opposition to geometric disparity. Depending on the relative phase of the geometric and either the contrast or luminance disparity, there may be a retinal illuminance disparity at the moment that the pixel turns on, which is a situation where the Pulfrich effect may occur. Roughly, there is about a 20 ms delay in processing for each log(Td) drop in retinal illuminance with a slowly-moving stimulus [31]. The largest luminance disparity for a bright bar during an edge shift for our stimuli implies a roughly 15 ms interocular delay in the perceived shift of the edge. Given that perceived depth due to geometric disparities vanishes at around 6 Hz [9,21,22,23,24,25,26,27], corresponding to 167 ms presentation time, the perceived effects of the Pulfrich delay would be negligible. Observers were asked to respond indicating whether or not the perceived rotation changed rapidly, or “jumped,” during the trial. It was anticipated that there would exist a phase where the perceived rotation caused by the contrast disparity would cancel the rotation caused by the geometric disparity, so the individual bars would appear to either rotate only slightly or be flat. Furthermore, it was imagined that, if the phase of the two were not aligned to give a continuous cancellation, then one disparity type would create rotation that would then be cancelled by the second disparity type, which produces a rapid drop in perceived rotation, or a “jump.” The probability of a rapid change in rotation was measured as a function of phase, or timing offset. Offsets were −200, −150, −100, −50, 0, 50, 100, 150, and 200 ms (The actual offsets were within +/− 0.5 (1000/75) = 6.7 ms of these nominal values.) and frequencies were again 0.2, 0.4, 0.6, and 0.8 Hz. Combining each frequency and offset gave 36 possible combinations. The experimental program was randomly selected from a combined set of 36 contrast combinations and 36 luminance combinations to create a total of 72 trials per experimental session. Each observer completed 20 sessions for a total of 20 choices at each frequency/offset combination.

Mathematica notebooks containing individual data files, models, and analyses can be downloaded as a .zip file from https://unh.box.com/v/dobiasstine2019data.

## 3. Results

**Figure 3:**
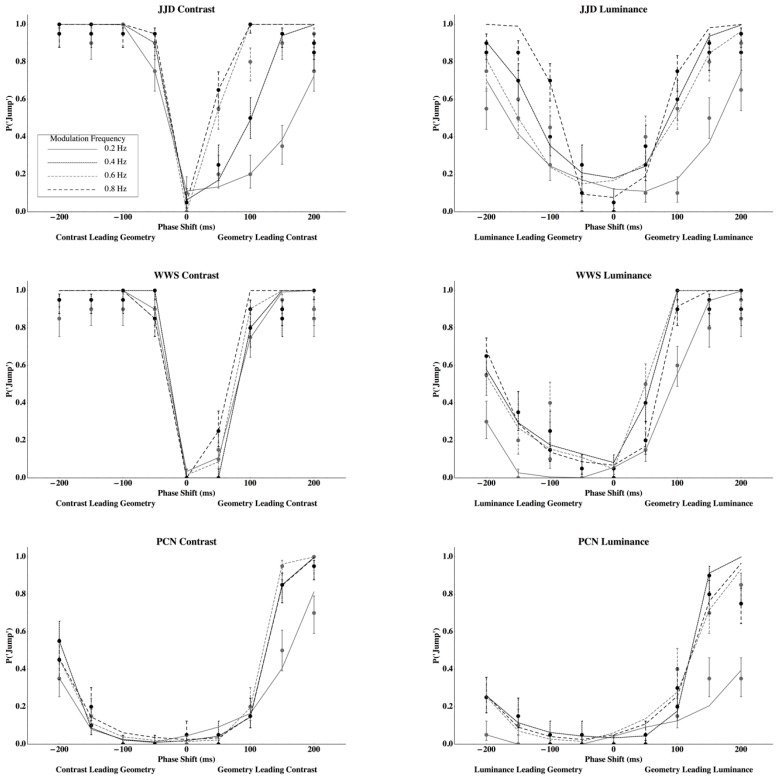
Data points with standard error bars describe the probability of reporting a “jump” for contrast (left) and luminance (right) disparity modulations for observer JJD (top), WWS (middle), and PCN (bottom). Standard errors are calculated using the score confidence interval (Agresti and Coull [32] (Equation (2)), Wilson, [33] with *n* = 20 and α = 0.318. Lines are the predicted probability of reporting a “jump” using the Gated Generalized Difference Model.

The Probability of reporting a jump for observers JJD, WWS, and PCN when viewing contrast and luminance disparities can be seen in Figure 3. 

Temporal offsets between contrast or luminance disparities and geometric disparities are plotted on the *x*-axes and the probabilities of reporting a jump are plotted on the *y*-axes. Individual points with standard error bars (score confidence intervals with *n* = 20 and α = 0.318, Agresti and Coull [32] (Equation (2)), Wilson [33]) represent measured probabilities and the lines connect probabilities that are predicted by the Gated Generalized Difference Model for the different modulation frequencies indicated in the legend (see Appendix B). Cancellation using contrast disparities are presented in the left column and those for luminance disparities in the right column. Each row represents the data for a given observer.

Comparing Figure 1 and Figure 3, results clearly support the Gated Generalized Difference Model over the Generalized Difference Model since the phase lag that minimizes the probability to perceive a jump is very close to zero for all conditions and observers (points with standard error bars in Figure 3). Predicted probabilities to perceive a jump from the gated model (lines in Figure 3) closely parallel those measured (see Table 1 for the coefficients of determination). To better understand the model’s fit, two parameters were used to fit each psychometric function from the gated model, and then an ANOVA was conducted using these parameters as dependent variables in order to determine the number of *unique* parameters required to describe the data.

In order to make individual-observer fits for the probability of a jump, the net difference in the predicted perceived rotation was passed between two geometric disparity modulations where one is cancelling the other through a discrete Fourier transformation, and calculated the mean and standard deviation of the maximum power across the spectrum and modulation frequencies at each lag. New parameters (means, *μ*, and standard deviations, *σ*) were then calculated in order to minimize the difference between the probability of perceiving a jump, as predicted from a Laplace cumulative density function (see Filley et al. [7]) and the observed probability at a particular lag as a function of the type of modulation (contrast or average luminance), the frequency of the modulation, the sign of the phase lag, and the observer (see Appendix B). Hence, two free parameters were used to fit each of the 48 psychophysical functions presented in Figure 3.

**Figure 4:**
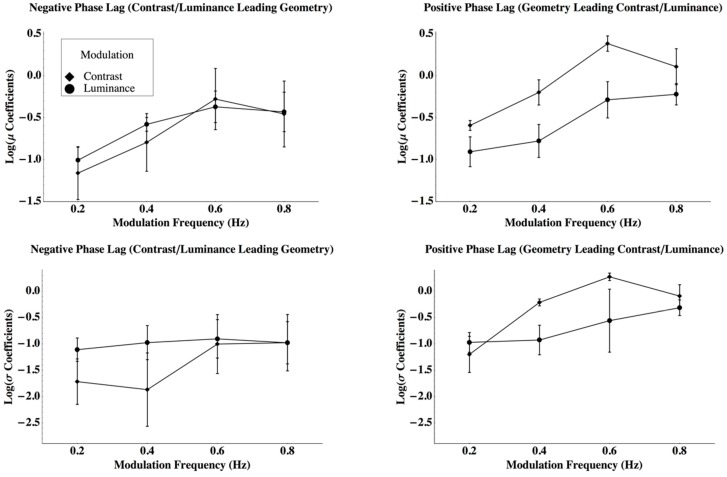
The parameters for the Laplace distributions represent response criteria to perceive a jump. The top row plots mean log (*μ*) and the bottom row mean log (*σ*) as a function of disparity modulation frequency with standard errors. The left column presents parameters for phase lags where contrast or average luminance disparities precede geometric disparity modulations and the right column where geometric modulations lead contrast or luminance modulations with standard errors.

The parameters for the Laplace distributions represent response criteria to perceive a jump, given that they describe the relationship between the power spectra of the net perceived rotation to the probability that a particular observer will respond with a ‘jump.’ Therefore, for example, suppose that an observer has a ‘high’ criterion. If so, he or she might only indicate a jump if it is very obvious. Their psychometric function will shift away from the origin, which is described as a change in the mean. If the coefficient of variation remained constant, the spread coefficient, which is positively related to the standard deviation, would increase. These parameters are presented in Figure 4. The top row plots mean log (*μ*) and the bottom row mean log (*σ*) as a function of disparity modulation frequency. The left column presents parameters for phase lags where contrast or average luminance disparities precede geometric disparity modulations and the right column where geometric modulations lead contrast or luminance modulations. To determine the number of *unique* parameters required to describe the data, a three-way repeated measures analysis of variance was conducted by using the parameters as dependent variables. The analysis suggested that the phase lag by the modulation type interaction was significant for both the central tendency, log (*μ*), and the spread, log (*σ*), parameters of the Laplace distribution (central tendency log (*μ*): *F*(1, 15) = 38.57, Giesser-Greenhouse adjusted *p* = 0.0125, partial ω^2^ = 0.758, *MSRES* = 0.147; spread log (β): *F*(1, 15) = 8.316, Giesser-Greenhouse adjusted *p* = 0.0384, partial ω^2^ = 0.454, *MSRES* = 0.758). Both the mean and standard deviation for the criterion power to perceive a jump was greater when a geometric disparity lead a contrast disparity than when a geometric disparity lead an average luminance disparity, or when either contrast or luminance disparities lead geometric disparities. Furthermore, there were differences among the observers with respect to the mean power required to detect a jump (*F*(2, 15) = 10.67, *p* = 0.0131, *ρ_I_* = 0.416, *MSRES* = 0.147). Other effects were not significant (see Appendix C). The adjusted coefficients of multiple determination were *R*^2^ = 0.812 and *R*^2^ = 0.520 for the mean and standard deviation criterion power, respectively [34]. Hence, to describe 16 psychometric functions measured for each observer, one mean and standard deviation are sufficient for the eight functions where contrast or luminance lead geometry, one mean and standard deviation when geometry leads contrast, and one mean and standard deviation when geometry leads average luminance (six parameters to describe 16 psychometric functions for each observer). Furthermore, the standard deviations do not differ among the observers even though the means do differ across observers (giving 16 parameters to describe 48 psychometric functions). In summary, the differences among the plots in Figure 3 may be fully described by differences in Laplace distribution means and standard deviations, which describe criterion effects in the model. Contrast has a slow onset, which creates larger means when contrast leads geometry, but a rapid offset, which gives means closer to zero when contrast leads the geometry. Both the mean and standard deviation for the criterion power to perceive a jump was greater when a geometric disparity led a contrast disparity than when a geometric disparity led an average luminance disparity or when either of the contrasts or luminance disparities led geometric disparities. The possibility of describing these differences with *gain* changes is discussed below.

## 4. Discussion

The experiment described above measured the dynamics of canceling perceived depth from a geometric disparity with the perceived depth resulting from a contrast or average luminance disparity. Based on the results from Dobias and Stine [9], we predicted that luminance and contrast disparities would have lower critical frequencies than geometric disparities. Furthermore, given the ability of observers to discriminate perceived rotation induced by a sine-wave contrast disparity modulation from that induced by a square-wave contrast disparity modulation [9], we predicted that the probability of a jump for relatively low frequencies would be minimized at a phase lag of nearly zero, as described by the Gated Generalized Difference Model. The results come from the three observers. While it is true that our reported data are based solely on responses from three observers (two of whom were authors), each observer did complete 20 sessions with each consisting of 72 trials. Overall, observers made a total of 20 choices at each frequency/offset combination. We believe that this extensive test of a smaller number of observers as opposed to a less extensive test of a larger number observers is advantageous and is consistent with acceptable experimental practices [35,36]. The observers’ results were consistent with these predictions. The results support the notion that the Venetian blind effect taps a form of stereopsis that is distinct from that due to geometric disparities. Irradiation models do not account for the effect.

Whether or not contrast and average luminance disparities tap distinct mechanisms is not clear. From the analysis of the Laplace coefficients, it was found that more power was required to perceive a jump when a geometric disparity modulation led a contrast modulation than when either a geometric led an average luminance modulation or either a contrast or luminance modulation led a geometric modulation. A similar pattern holds for the slope of the psychometric function relating power to the probability to perceive a jump. The difference between the case when geometry modulations lead contrast modulations and the remaining three possibilities may be due to some form of higher-level processing reflected in criterion shifts, which is how these effects were described within the model or, to more fundamental, lower level, differences between, perhaps, contrast and average luminance channels feeding into the cyclopean locus. Given the ability to describe these different psychometric functions using criterion differences, inferring separate channels for contrast and average luminance based on the described data would seem unparsimonious.

Furthermore, based on the work of Sclar et al. [37], Geisler et al. [38], and Hetley and Stine [8] developed a form of the Naka-Rushton equation that may describe the behavior of cortical cells to changes in both the contrast and average luminance of a stimulus (Reference [8], Equations (13) and (14)). Hetley and Stine then used that equation in a difference model to describe perceived rotation under both contrast and average luminance disparities. It is certainly plausible, then, that contrast and average luminance disparities do share a common channel or mechanism, which implies that the significant phase lag by modulation type interaction for the Laplace parameters is due to some form of higher-level processing reflected in criterion shifts. More work is needed to resolve this question.

What these channels and locus represent anatomically is also unclear. Livingstone and Hubel [39] argued that a pathway for stereo and motion information through magnocellular layers of the lateral geniculate nucleus maps to layers 4Cα and 4B of the visual cortex area V1, then to the thick cytochrome oxidase-defined stripes in area V2, and on to area V5 (middle temporal area MT). Hence, one might be tempted to infer that the contrast and average luminance effects that were measured operate through the magnocellular pathway.

However, at least four lines of evidence argue against this hypothesis. As described by Sincich and Horton [40]: “First, [V1] layer 4B gets both parvo and magno input [41]. Second, lesions of magnocellular geniculate laminae have no effect on stereopsis [42]. Third, disparity-tuned cells are abundant outside [V1] layer 4B [43] and thick stripes [in V2] [44,45]. Fourth, other layers besides [V1 layer] 4B project to [area V2] thick stripes [46].” Furthermore, inputs from the magnocellular, parvocellular, and koniocellular layers of the lateral geniculate nucleus are intermixed within V1 [46], which, perhaps, suggests that the parallel streams that these inputs represent vanish.

Current evidence suggests that binocular cells in V1 respond to absolute disparities [47] while many in V2 also respond to relative disparities [48]. Beyond V2, several areas of the cortex respond to specific forms of disparities [49], which suggests that stereopsis in various forms is widely distributed across the cortex. Furthermore, reciprocal connections abound. The projections from V2 to V1 are nearly 80% as numerous as those from V1 to V2 [50]. Hence, there is little upon which to base speculation about the anatomy of the contrast/luminance channels, gating, or the cyclopean locus.

## Figures and Tables

**Table 1:** Coefficients of determination for modulations of contrast and luminance disparity for observers JJD, WWS, and PCN.

Observer	Modulation Type	*R* ^2^	Adjusted *R*^2^
JJD	Contrast	0.995	0.991
	Luminance	0.974	0.954
WWS	Contrast	0.994	0.989
	Luminance	0.984	0.973
PCN	Contrast	0.993	0.987
	Luminance	0.971	0.949

## Appendix A. The Dynamic Models of the Venetian Blind Effect

### Appendix A.1. Generalized Difference Model

The Generalized Difference Model, which is fully described by Dobias and Stine [9], effectively states that the perceived rotation varies with the generalized interocular difference in the neural response to average luminance or contrast between the two eyes. The neural response to contrast was described by the Naka-Ruston [51] equation where *n* = 1 and *M* = 0 [9,35].

(A1) $$R\left(C\right)=\frac{R_{max}C^{n}}{C^{n}+\sigma_{50}^{n}}+M$$

where *R_max_ =* 27.4 imp/s is the maximum response rate for the cell, *C* is the contrast of a grating, σ_50_ = 0.15 is the semi-saturation constant, or that contrast that gives a response for the cell that is half *R_max_*, *M =* 8.22 imp/s is the resting response rate of the cell, and *n* = 2.4. The perceived edge shift in response to a contrast disparity was modeled using a system of three differential equations [9] (System 1).

(A2) $$\{\begin{array}{c} \frac{df_{l}\left(t\right)}{dt}=\frac{1}{\tau }\left(-f_{l}\left(t\right)+R\left(C_{l}\left(t\right)\right)\right)\\ \frac{df_{r}\left(t\right)}{dt}=\frac{1}{\tau }\left(-f_{r}\left(t\right)+R\left(C_{r}\left(t\right)\right)\right)\\ \frac{dg\left(t\right)}{dt}=\frac{1}{\tau_{cyc}}\left(-g\left(t\right)+gain\left(f_{l}\left(t\right)-f_{r}\left(t\right)\right)\left(f_{l}\left(t\right)-M\right)\left(f_{r}\left(t\right)-M\right)\right) \end{array}$$

where the contrast of the grating presented to a particular eye, *C_eye_*(*t*), changes as a function of time, *R*(*C*_eye_(*t*)) is Equation (A1) and describes the steady-state response of a cell to contrast, *f_eye_*(*t*) describes the dynamic neural response to the contrast presented to a given eye, incorporating *R*(*C*_eye_(*t*)), τ is the time constant for the neural response to contrast for either eye, *g*(*t*) is the cyclopean perceived disparity of the particular edges of the bars composing the rectangular-wave grating, *M* is defined with respect to Equation (A1), *gain* is the parameter controlling the strength of the input from the generalized difference in a neural response to the grating pairs, and τ*_cyc_* is the time constant for the cyclopean response. One of the solutions to System (A2), *g*(*t*) (see Equation (3) in Dobias and Stine [9]), is used to calculate the perceived horizontal size ratio (Dobias and Stine [9], Equation (4)).

(A3) $$PercHSR\left(C_{l}\left(t\right),C_{r}\left(t\right)\right)=\frac{width+2g\left(t\right)}{width-2g\left(t\right)}\;$$

where *width* = 0.4° of the retinal angle and is the width of each light bar of the rectangular wave grating when the light bars are perceived as the figure. The perceived horizontal size ratio is related to perceived rotation [52] (Equation (A5)), which is repeated in Equation (B1) in Dobias & Stine [9].

### Appendix A.2. Gated Generalized Difference Model

The gated model from Dobias and Stine [9] defined the perceived edge shift, *p*(*t*), as the product of the inter-ocular generalized difference in contrast and the magnitude of the Generalized Difference Model integrated over a single period of the stimulus at the critical frequency, *ω* Hz. Therefore, using the solutions from System (1), *f_eye_*(*t*) for the left and right eyes with *g*(*t*),

(A4) $$p\left(t\right)=gain\left(f_{l}\left(t\right)-f_{r}\left(t\right)\right)\left(f_{l}\left(t\right)-M\right)\left(f_{r}\left(t\right)-M\right)\int_{t-\frac{1000}{\omega }}^{t}\left|g\left(t^{\prime }\right)\right|dt^{\prime }\;$$

The values for *gain* and *M* were defined previously.

To summarize, the Generalized Difference Model describes a perceived edge shift from a solution to System (A2), *g*(*t*), and the resulting perceived horizontal size ratio change using Equation (A3) while the Gated Generalized Difference Model describes the perceived edge shift from Equation (A4) and the resulting perceived horizontal size ratio change from Equation (A3) using *p*(*t*) instead of *g*(*t*).

## Appendix B. Predictions and Fits from the Dynamic Models

For both the generalized difference and the Gated Generalized Difference Models, a four second interval from a 10-second trial was described where, at zero ms, two superimposed gratings were presented to the model with zero disparity. Disparity was then modulated for the two gratings to induce rotations in opposite directions. One grating was presented with a geometric disparity and the other with either an average luminance or a contrast disparity. Net perceived rotation, *r*(*t*), was defined as the difference in perceived rotation between the two superimposed gratings, calculating perceived rotation using either the Generalized Difference Model or the Gated Generalized Difference Model. Perceived rotation from the geometric disparity was modeled from the horizontal size ratio (Equation (A5), in Backus et al. [52]) with perceived edge movement dynamics using System (A2) setting τ = τ*_cyc_*/100 (after Dobias and Stine [9]) coupled with, if appropriate, Equation (A4). Perceived rotation from the luminance or contrast disparity was modeled similarly, but using τ equal to 79.95 times that used for the geometric modulation (corresponding to the average measured by Dobias and Stine [9]). The relative phases of the disparity modulations were varied over the range used in the experiment. The *Mathematica* 8 routine *NDSolve* was used for the numerical simulations of System (A2). Again, details for choosing constants are outlined in Appendix B of Dobias and Stine [9].

To define the probability of perceiving a jump, the net difference in perceived rotation of a geometric modulation canceling another geometric modulation was passed through a discrete Fourier transformation, *F*(*r*(*t*)) = *F*(ω). This was completed for phases ranging from −200 ms to 200 ms in 50-ms steps and modulation frequencies of 0.2, 0.4, 0.6, and 0.8 Hz during the interval 1 s to 4 s in a stimulus presentation. The mean and standard deviation of the maximum power, ${\left|F\left(\omega \right)\right|}^{2}$, was then calculated across the spectrum and modulation frequencies at each lag. A Laplace cumulative density function with the calculated mean and standard deviation was used to define the probability of perceiving a jump at a particular lag.

Lastly, the predictions for Figure 1 were derived by simulating a geometric modulation presented with either a contrast or average luminance modulation while applying the Laplace distribution from the geometric/geometric case.

The general predictions were fit to individual data by allowing the central tendency and spread of the Laplace distributions to vary as a function of the type of modulation (contrast or average luminance), the frequency of the modulation, the sign of the phase lag, and the observer. Hence, two parameters were used to fit each of the 48 psychophysical functions.

## Appendix C. Analysis of Variance for the Laplace Parameter Estimates

An analysis of variance using a three-factorial, repeated-measures model with a sign of the phase lag (contrast/luminance leading geometry, geometry leading contrast/luminance), modulation frequency (0.2, 0.4, 0.6, and 0.8 Hz), and modulation type (contrast, luminance) across three observers (JJD, WWS, and PCN) for each of two dependent variables, log mean, *μ*, and log spread, *β* = 2^−1/2^ *σ* (RBF-242, [34], Ch. 10). A Holm’s sequentially rejected procedure based on the Šidák inequality [53] and [34] was used to hold the family-wise Type I error to *α* = 0.05 across the two dependent variables.

Both dependent variables appeared normally distributed with no outliers even though the two omnibus variance-covariance matrices did appear non-spherical (log (*μ*) Giesser-Greenhouse *ε* = 0.1998, log (*β*) Giesser-Greenhouse *ε* = 0.2807, [34]). Furthermore, there was no evidence of an observer by treatment interaction for log (*μ*) (Tukey’s test of non-additivity: *F*(1, 29) = 0.748, *p* > 0.10, *MSRES* = 0.064, [34]) even though there was a treatment by observers’ interaction for log (*β*) (*F*(1, 29) = 7.319, *p* = 0.011, *MSRES* = 0.247). Hence, for log (*β*), one might anticipate reduced power and, for any significant effects, that effect cannot be generalized to all three observers.

Preliminary tests at *α* = 0.25 suggested that the three-way interaction, the phase lag by modulation frequency interaction, and the modulation frequency by the modulation type interaction should be pooled into the residual for both dependent variables [34] (Sec. 9.11). Again, using the Holm’s sequentially rejected procedure with Giesser-Greenhouse adjusted *p*-values, only the phase lag by modulation type interaction was significant (log (*μ*): *F*(1, 15) = 38.57, Giesser-Greenhouse adjusted *p* = 0.0125, partial *ω*^2^ = 0.758, *MSRES* = 0.147; log (*β*): *F*(1, 15) = 8.316, Giesser-Greenhouse adjusted *p* = 0.0384, partial *ω*^2^ = 0.454, *MSRES* = 0.758). Phase lag (log (*μ*): *F*(1, 15) = 8.544, Giesser-Greenhouse adjusted *p* > 0.05, *MSRES* = 0.147; log (*β*): *F*(1, 15) = 3.842, *p* > 0.05, *MSRES* = 0.758), modulation frequency (log (*μ*): *F*(1, 15) = 10.14, Giesser-Greenhouse adjusted *p* > 0.05, *MSRES* = 0.147, log (*β*): *F*(1, 15) = 1.788, *p* > 0.05, *MSRES* = 0.758), and modulation type (log (*μ*): *F*(1, 15) = 3.234, Giesser-Greenhouse adjusted *p* > 0.05, MSRES = 0.147, log (*β*): *F*(1, 15) = 7.548 × 10^−4^, *p* > 0.05, *MSRES* = 0.758) showed no effects for either dependent variable. There were differences among the observers for log (*μ*) (*F*(2, 15) = 10.67, *p* = 0.0131, *ρ_I_* = 0.416, *MSRES* = 0.147) but not log (*β*) (*F*(2, 15) = 3.514, *p* > 0.05, *MSRES* = 0.758).
